# Supplementary figures and images for: Monitoring deforestation, forest health, and environmental criticality in a protected area periphery using Geospatial Techniques
Source: PeerJ. 2024 Jul 18;12:e17714. doi: 10.7717/peerj.17714 (PMC11260410; doi:10.7717/peerj.17714)

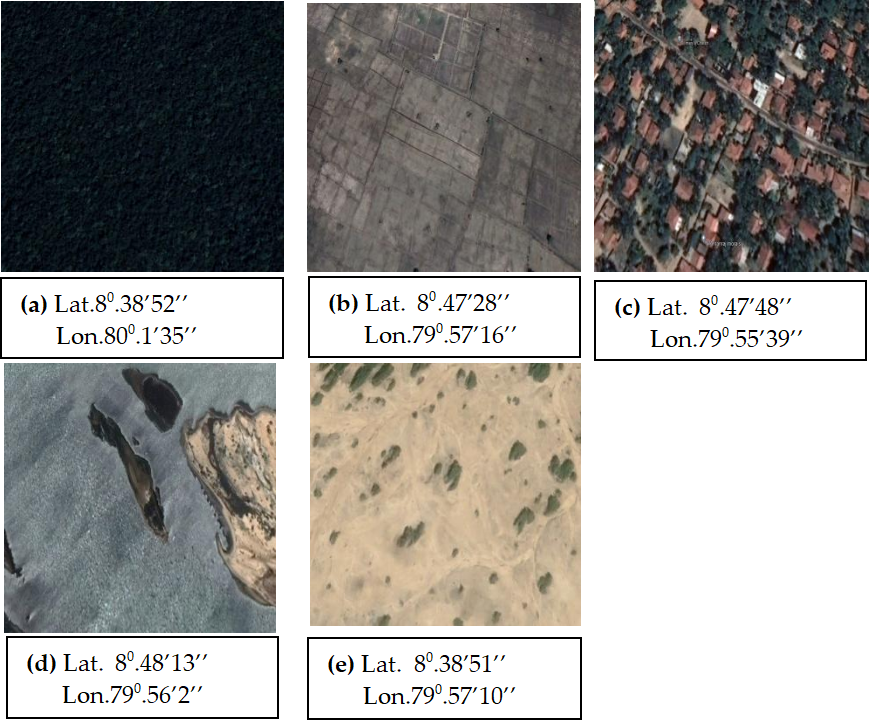

Supplement: Supplemental Information 2 [file peerj-12-17714-s002.png]

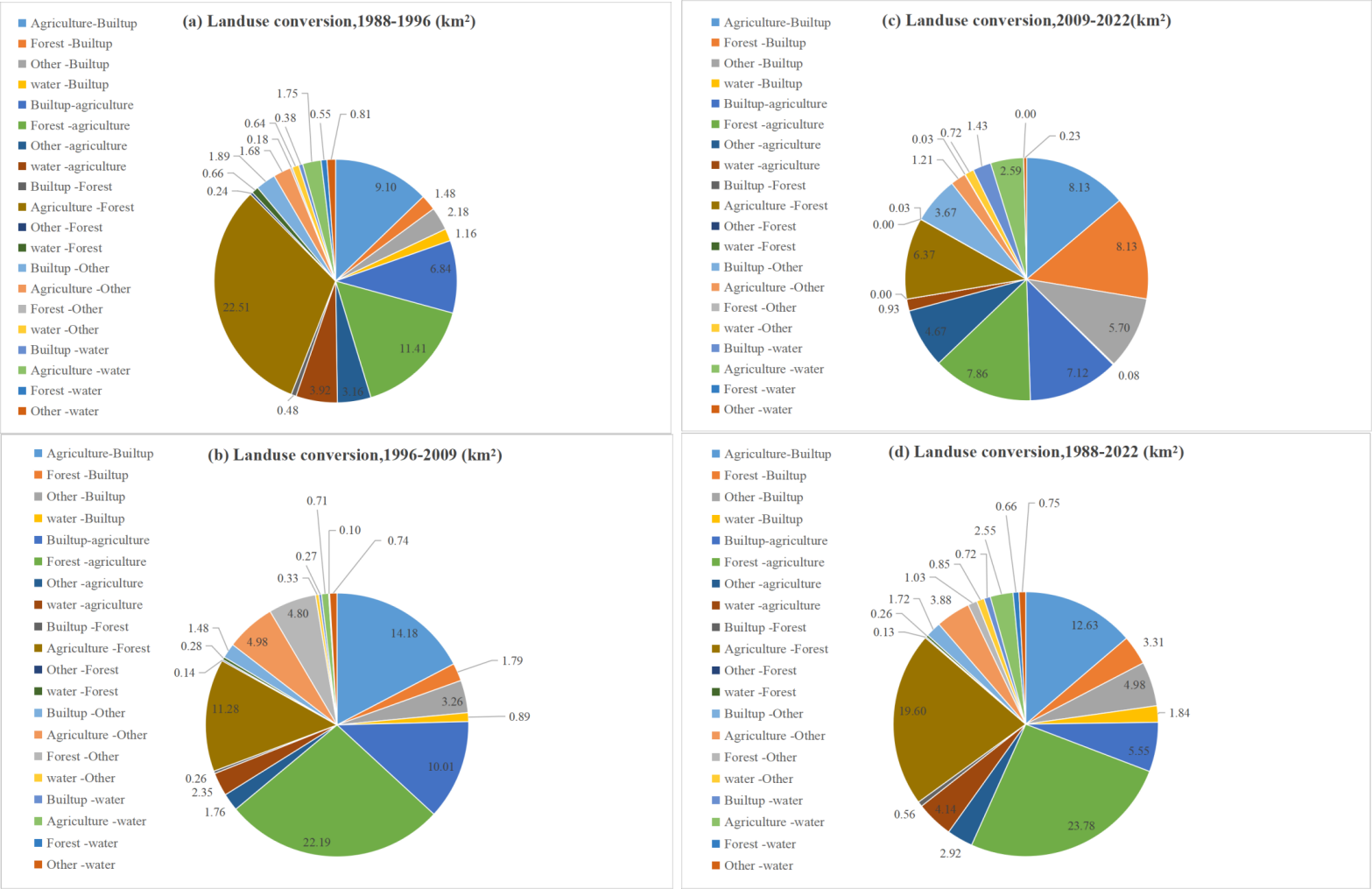

Supplement: Supplemental Information 3 [file peerj-12-17714-s003.png]

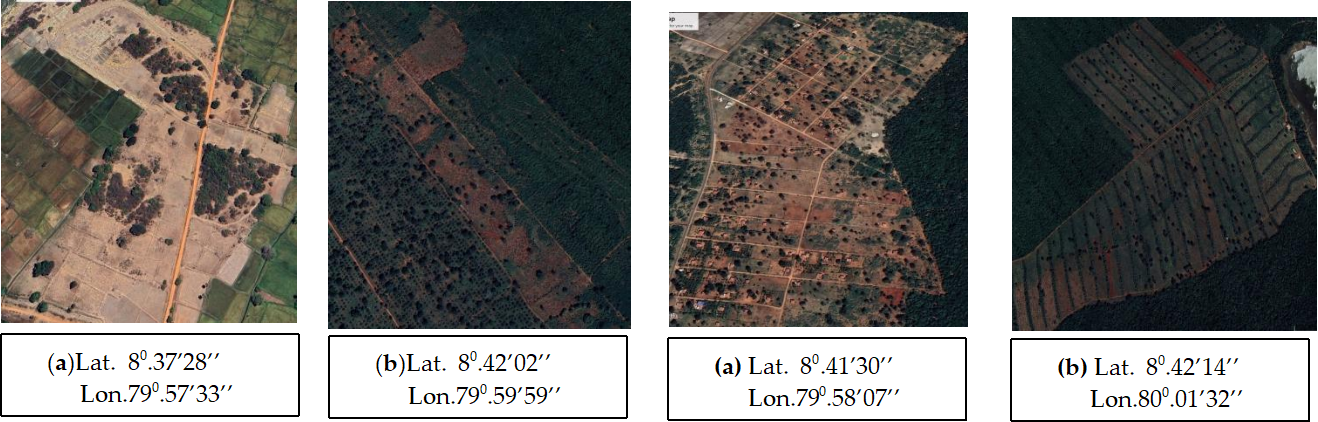

Supplement: Supplemental Information 4 [file peerj-12-17714-s004.png]

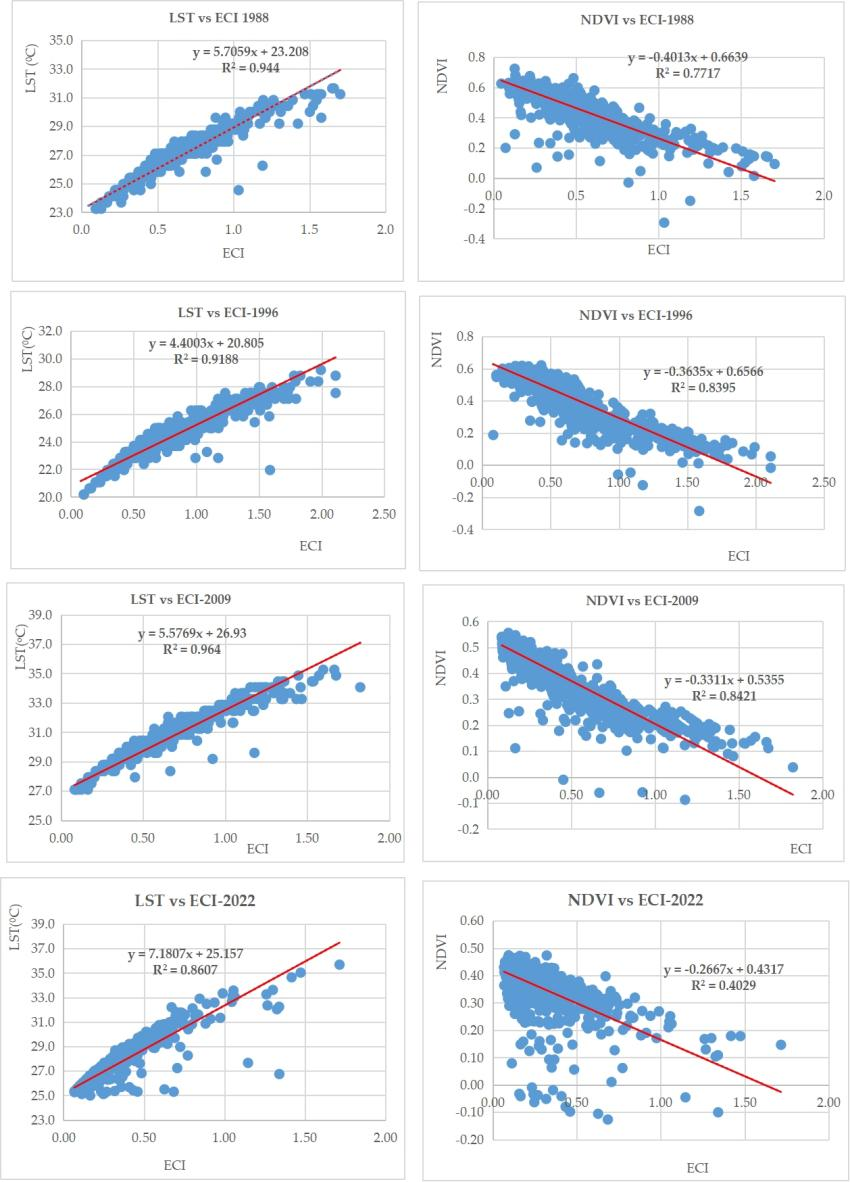

Supplement: Supplemental Information 5 [file peerj-12-17714-s005.png]

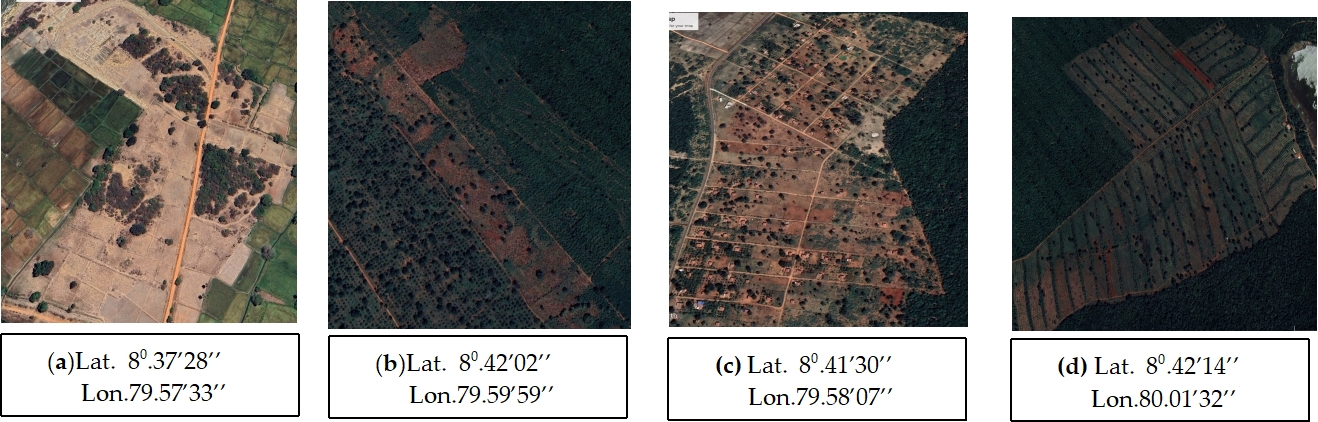

Supplement: Supplemental Information 6 [file peerj-12-17714-s006.png]

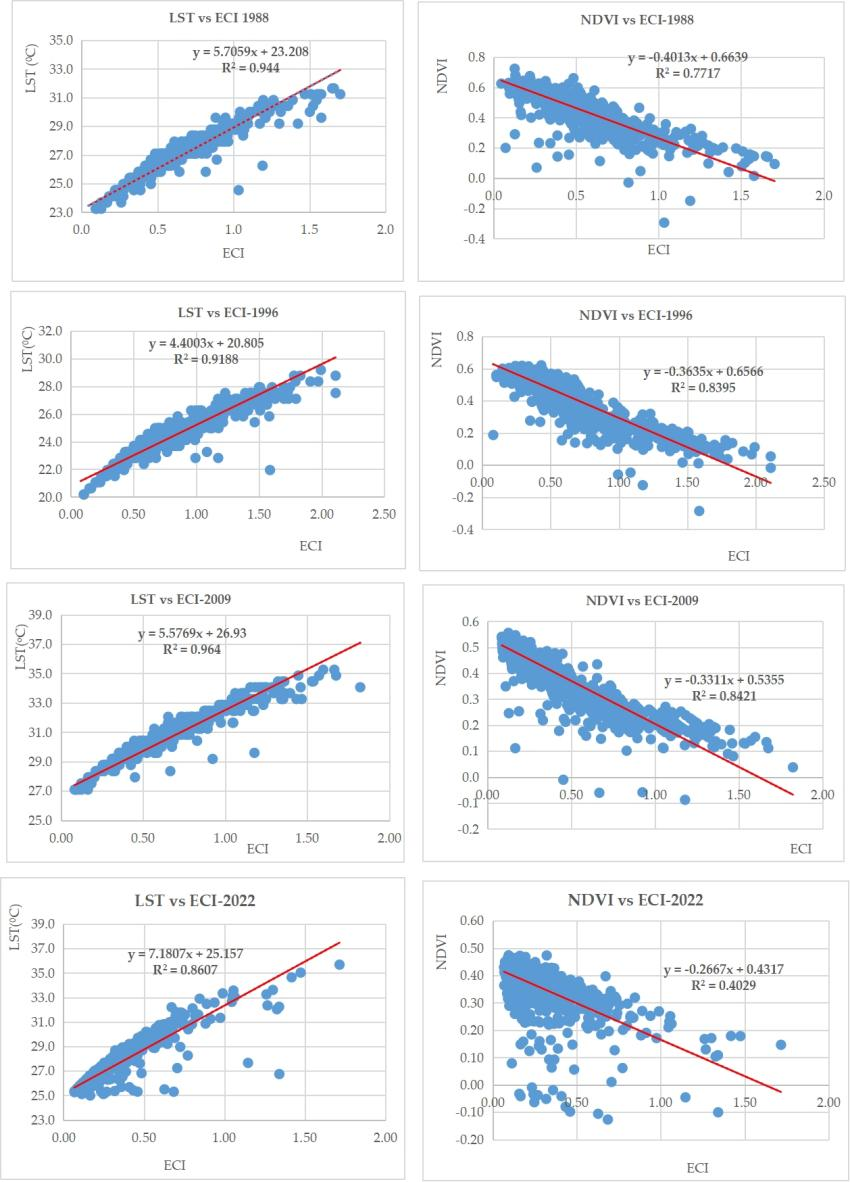

Supplement: Supplemental Information 7 [file peerj-12-17714-s007.png]
