# Supplementary material for: Monitoring deforestation, forest health, and environmental criticality in a protected area periphery using Geospatial Techniques
Source: PeerJ. 2024 Jul 18;12:e17714. doi: 10.7717/peerj.17714 (PMC11260410; doi:10.7717/peerj.17714)
Supplement: Supplemental Information 8 [file peerj-12-17714-s008.docx]

**Table S1**

Specifications of Landsat data used for the study.

| **Satellite** | **Sensor** | **Path/Row** | **Year** | **Resolution(m)** | **Wavelength (µm)** |
| --- | --- | --- | --- | --- | --- |
| Landsat-5 | Thematic  Mapper(TM) | 142/54 | 1988  1996  2009 | 30 | (B 1)0.45–0.52  (B 2)0.52–0.60  (B 3)0.63–0.69  (B4)0.76–0.90  (B 5)1.55–1.75  (B 6)10.40–12.50  (B 7)2.09–2.35 |
| Landsat-8 | Operational  Land Images  (OLI) and  Thermal  Infrared  Sensor  (TIRS) | 142/54 | 2022 | 30 | (B1)0.43–0.45  (B2)0.45–0.51  (B 3)0.53–0.59  (B 4)0.64–0.67  (B5)0.85–0.88  (B 6)1.57–1.65  (B 7)2.11–2.29  (B 8)0.50–0.68  (B 9)1.36–1.38  (B10)10.60–11.19  (B11)11.50–12.51 |
