# Supplementary material for: Monitoring deforestation, forest health, and environmental criticality in a protected area periphery using Geospatial Techniques
Source: PeerJ. 2024 Jul 18;12:e17714. doi: 10.7717/peerj.17714 (PMC11260410; doi:10.7717/peerj.17714)
Supplement: Supplemental Information 9 — *Image reference with respect to Fig. S1 [file peerj-12-17714-s009.docx]

**Table S2**

LULC classes with codes and descriptions.

| **LULC** | **Code** | **Description** | **Image reference*** |
| --- | --- | --- | --- |
| Forest cover | FC | High and low density  forests | a |
| Agriculture | AG | Irrigated /rain-fed paddy cultivation areas and plantations | b |
| Buildup/settlements | BS | Impervious and residential lands | c |
| Water bodies | WB | Areas covered by  streams, tanks, and ponds | d |
| Other | OT | Extensive sand surfaces | e |

*Image reference with respect to Figure S1.
