# Supplementary material for: Monitoring deforestation, forest health, and environmental criticality in a protected area periphery using Geospatial Techniques
Source: PeerJ. 2024 Jul 18;12:e17714. doi: 10.7717/peerj.17714 (PMC11260410; doi:10.7717/peerj.17714)
Supplement: Supplemental Information 10 — FC, Forest cover; AG, Agriculture; BS, Built-up/settlements; WB, Water bodies; OT, Others [file peerj-12-17714-s010.docx]

**Table S3.**

Statistics of accuracy assessments of LULC classes.

|  | **LULC** | **1988** | **1996** | **2009** | **2022** |
| --- | --- | --- | --- | --- | --- |
| User accuracy (%) | FC | 99 | 98 | 98 | 96 |
|  | AG | 96 | 88 | 82 | 78 |
|  | BS | 74 | 71 | 78 | 64 |
|  | WB | 98 | 96 | 92 | 90 |
|  | OT | 92 | 88 | 82 | 78 |
| Producer accuracy (%) | FC | 100 | 94 | 92 | 94 |
|  | AG | 94 | 82 | 78 | 74 |
|  | BS | 71 | 70 | 74 | 60 |
|  | WB | 92 | 91 | 88 | 86 |
|  | OT | 88 | 80 | 80 | 74 |
| Overall accuracy(%) and Kappa |  | 84 | 81.8 | 80.1 | 79.3 |
|  |  | 0.79 | 0.77 | 0.76 | 0.75 |

FC, Forest cover; AG, Agriculture; BS, Built-up/settlements; WB, Water bodies; OT, Others.
