# Supplementary material for: Monitoring deforestation, forest health, and environmental criticality in a protected area periphery using Geospatial Techniques
Source: PeerJ. 2024 Jul 18;12:e17714. doi: 10.7717/peerj.17714 (PMC11260410; doi:10.7717/peerj.17714)
Supplement: Supplemental Information 11 [file peerj-12-17714-s011.docx]

**Table S4**

Descriptive statistics of NDVI changes in Musali DSD.

| **Date** | Min | Max | Mean | St.Deviation |
| --- | --- | --- | --- | --- |
| 6 February 1988 | -0.55 | 0.75 | 0.51 | 0.12 |
| 4 August 1996 | -0.48 | 0.85 | 0.44 | 0.14 |
| 3 March 2009 | -0.33 | 0.58 | 0.43 | 0.10 |
| 3 February 2022 | -0.18 | 0.54 | 0.37 | 0.07 |
