# Supplementary material for: Monitoring deforestation, forest health, and environmental criticality in a protected area periphery using Geospatial Techniques
Source: PeerJ. 2024 Jul 18;12:e17714. doi: 10.7717/peerj.17714 (PMC11260410; doi:10.7717/peerj.17714)
Supplement: Supplemental Information 12 [file peerj-12-17714-s012.docx]

**Table S5**

Descriptive statistics of retrieved LST changes in Musali DSD (^0^C).

| **Date** | Min | Max | Mean | St.Deviation |
| --- | --- | --- | --- | --- |
| 6 February 1988 | 22.3 | 32.8 | 25.2 | 1.57 |
| 4 August 1996 | 19.2 | 30.4 | 23.3 | 1.70 |
| 3 March 2009 | 26.2 | 36.8 | 28.6 | 1.72 |
| 3 February 2022 | 24.5 | 38.8 | 26.6 | 1.38 |
